# Vergleich der DRG-Erlöse zwischen Fast- und Slow-Track-Verfahren beim zweizeitigen Prothesenwechsel bei periprothetischen Hüftinfektionen im aG-DRG-System 2020

**DOI:** 10.1007/s00132-021-04106-8

**Published:** 2021-04-21

**Authors:** Katja Hierl, Markus Rupp, Michael Worlicek, Florian Baumann, Christian Pfeifer, Volker Alt

**Affiliations:** Klinik und Poliklinik für Unfallchirurgie, Universitätsklinikum Regensburg (UKR), Franz-Josef-Strauß-Allee 11, 93053 Regensburg, Deutschland

**Keywords:** Diagnosis Related Group, Krankenhaus, Aufenthaltsdauer, Revisionschirurgie, Staphylococcus-Infektion, Diagnosis Related Group, Hospitals, Length of stay, Revision surgery, Staphylococcal infections

## Abstract

**Hintergrund:**

Die Behandlung periprothetischer Hüftinfektionen ist meist kostenintensiv und gilt im Allgemeinen als nicht kostendeckend für die Kliniken. Bei chronischen Infektionen ist oft ein zweizeitiger Prothesenwechsel indiziert, der als Fast-Track mit kurzem prothesenfreiem Intervall (2–4 Wochen) oder als Slow-Track mit langem prothesenfreiem Intervall (über 4 Wochen) erfolgen kann.

**Ziel:**

Ziel dieser Arbeit war die Erfassung der Erlössituation beider Behandlungsformen im aktuellen aG-DRG-System 2020 unter Berücksichtigung erlösrelevanter Einflussfaktoren.

**Methoden:**

Für Fast-Track und Slow-Track bei zweizeitigem septischem Hüftprothesenwechsel mit Nachweis eines Staphylococcus aureus (MSSA) wurden mittels einer Grouper-Software (3M KODIP Suite) anhand der Diagnosen (ICD-10-GM) und Prozeduren (OPS) Behandlungsfälle simuliert und in DRG eingruppiert. Erlösrelevante Parameter wie Verweildauer (VWD) und Nebendiagnosen (ND) wurden berücksichtigt. Zusätzlich wurden zwei reale Behandlungsfälle mit Fast-Track und Slow-Track miteinander verglichen.

**Ergebnisse:**

Die Gesamterlöse betrugen beim Slow-Track bei einer VWD von 25 Tagen (ohne ND) 27.551 € und bei einer VWD von 42 Tagen (mit ND) 40.699 €. Beim Fast-Track hingegen lag der Gesamterlös bei 23.965 € bei einer VWD von 25 Tagen (ohne ND) und bei 27.283 € bei einer VWD von 42 Tagen (mit ND). Bei den realen Behandlungsfällen zeigte sich ebenfalls eine deutliche Differenz des Gesamterlöses von 12.244 € zugunsten des Slow-Tracks.

**Diskussion:**

Auch im aG-DRG-System 2020 scheint der zweizeitige Hüftprothesenwechsel mit langem Interimsintervall insbesondere bei multimorbiden Patienten aus Krankenhaussicht ökonomisch vorteilhafter zu sein als das Fast-Track-Konzept, wodurch ein finanzielles Hemmnis zur Behandlung solcher Patienten mit kurzem Interimsintervall geschaffen wird.

## Abstract (en)

**Background:**

The treatment of periprosthetic hip infections is usually cost intensive, so it is generally not cost effective for hospitals. In chronic infections, a two-stage procedure is often indicated, which can be done as a fast-track procedure with a short prosthetic-free interim interval (2–4 weeks) or as a slow-track procedure with a long prosthetic-free interim interval (over 4 weeks).

**Aim:**

The aim of this study was to elucidate the revenue situation of both forms of treatment in the aG-DRG-System 2020, taking into account revenue-relevant influencing factors.

**Methods:**

For fast-track and slow-track procedures with two-stage revision and detection of a staphylococcus aureus (MSSA), treatment cases were simulated using a grouper software (3M KODIP Suite) based on the diagnoses (ICD-10-GM) and procedures (OPS) and then grouped into DRGs. Revenue-relevant parameters, such as length of stay and secondary diagnoses (SD), were taken into account. In addition, two real treatment cases with fast-track and slow-track procedures were compared to each other.

**Results:**

The total revenues for the slow-track procedure with a length of stay of 25 days (without SD) were 27,551 € and for a length of stay of 42 days (with SD) even 40,699 €, compared to 23,965 € with the fast-track procedure with a length of stay of 25 days (without SD) and 27,283 € for a length of stay of 42 days (with SD). The real treatment cases also showed a big difference in the total revenues of 12,244 € in favor of the slow-track procedure*.*

**Discussion:**

Even in the aG-DRG-System 2020, the two-stage revision procedure with a long interim interval seems to be more interesting from a financial point of view and the hospital perspective compared to the fast-track procedure, especially with multimorbid patients. This creates a financial barrier to the treatment of such patients with a short interim interval.

## Hintergrund und Fragestellung

Durch das DRG-System wird in allen Bereichen der Medizin eine Schaffung von Fehlanreizen beobachtet, indem bestimmte diagnostische und operative Maßnahmen hoch vergütet bzw. der Anreiz zur Erlössteigerung vermittelt werden [5]. Die vorliegende Arbeit widmet sich der Analyse der neuen Abrechnungsmodalitäten nach dem aG-DRG-System (ausgegliedertes G‑DRG-System), das zum 01. Januar 2020 eingeführt wurde, für chronische periprothetische Hüftinfektionen mit der Frage, ob auch hier ökonomische Anreize für ein bestimmtes Therapieregime bestehen.

Periprothetische Hüftinfektionen sind schwerwiegende und medizinisch sowie ökonomisch herausfordernde Komplikationen in der Endoprothetik [1, 6, 22, 26], deren Häufigkeit nach primärer Prothesenimplantation mit 0,5–2 % und bei Hüftprothesenrevisionen mit 4 % angegeben wird [8, 10, 28]. Bei weltweit zunehmender Anzahl an Primärimplantationen von Hüftprothesen ist von einem Anstieg periprothetischer Infektionen in den nächsten Jahren auszugehen [12, 25]. Entsprechend der demografischen Entwicklung sind die betroffenen Patienten häufig in hohem Lebensalter und haben zahlreiche Komorbiditäten, sodass die Verläufe periprothetischer Hüftinfektionen meist komplex und mit einer hohen Morbidität und Mortalität verbunden sind [13, 23, 24, 29].

In Abhängigkeit von der zugrundeliegenden Ausgangssituation ist bei periprothetischen Hüftinfektionen oft ein Prothesenwechsel erforderlich, der in der Regel zweizeitig mit langem prothesenfreiem Intervall von mindestens 4 Wochen (Slow-Track) durchgeführt wird. Alternativ kann der Prothesenwechsel als Fast-Track mit kurzem prothesenfreien Intervall (2–4 Wochen) durchgeführt werden, falls dies von Seiten der Knochen- und Weichteilsituation sowie des Keim- und Resistenzmusters möglich ist [16, 19]. Voraussetzung hierfür ist die Abwesenheit eines Problemkeims, der nicht mit biofilmgängigen Antibiotika, wie z. B. Rifampicin oder Ciprofloxacin, therapiert werden kann. Generell wird ein Fast-Track-Konzept aufgrund des besseren Gewebezustandes bei der Prothesenreimplantation, der schnelleren Mobilisation und Verkürzung der Gesamtbehandlungsdauer als vorteilhaft angesehen [16]*.*

Im Allgemeinen sind die Behandlungen periprothetischer Hüftinfektionen sehr kostenintensiv und gelten als nicht kostendeckend, sodass sie für die Kliniken ein wirtschaftliches Problem darstellen [3, 4, 9, 21]. Daher spielen bei der Planung der stationären Behandlung beim zweizeitigen Prothesenwechsel neben medizinischen Gründen zunehmend auch ökonomische Aspekte eine Rolle, die sich aus dem G‑DRG-System mit pauschaliertem Abrechnungsverfahren ergeben. Erfolgt die Wiederaufnahme nach Entlassung aus dem ersten stationären Aufenthalt bei gleicher Basis-DRG innerhalb der oberen Grenzverweildauer oder 30 Kalendertage ab Aufnahmetag des ersten Aufenthaltes, führt dies laut Fallpauschalenvereinbarung (§2) zu einer Fallzusammenführung. Hierdurch kommt es gerade bei der Behandlung äußerst kostenintensiver periprothetischer Hüftinfektionen häufig zu ökonomischen Fehlanreizen, indem der zweizeitige Prothesenwechsel mit entsprechend langem Intervall geplant wird, um eine Fallzusammenlegung zu umgehen und zwei stationäre Fälle abrechnen zu können. Eine Fast-Track-Behandlung mit einem oder zwei stationären Aufenthalten mit kurzem Intervall von 2–4 Wochen ist für die Kliniken demzufolge meist mit großen Erlösverlusten verbunden [20]. Jedoch kann dieses Vorgehen insbesondere bei multimorbiden Patienten, deren Versorgung im prothesenfreien Intervall sich häufig schwierig gestaltet, einen geeigneten Behandlungsweg darstellen. Aus wirtschaftlicher Sicht ist dieses Konzept allerdings nachteilig für die Kliniken, da solche Fälle mit einem stationären Aufenthalt bzw. bei einer Fallzusammenlegung aufgrund des kurzen Intervalls im G‑DRG-System nur unzureichend abgebildet werden [20].

Gemäß Pflegepersonal-Stärkungsgesetz wurde zum 01. Januar 2020 die „Pflege am Bett“ aus der Finanzierung über G‑DRG ausgegliedert und das aG-DRG-System eingeführt, bei dem der Fallpauschalenkatalog um den Pflegeerlöskatalog erweitert wurde. Die Erstattung der Pflegekosten erfolgt ab 2020 über ein Pflegebudget per Selbstkostendeckungsprinzip. Bis zur Verhandlung des krankenhausindividuellen Pflegebudgets wurde der tagesbezogene Pflegeentgeltwert zum 01. Januar 2020 auf 146,55 € festgesetzt und aufgrund der Coronavirus-Pandemie zum 01. April 2020 bis 31. Dezember 2020 auf 185 € erhöht, gemäß COVID-19-Krankenhausentlastungsgesetz [7].

Vor diesem Hintergrund sollte die Erlössituation von Fast-Track und Slow-Track beim zweizeitigen septischen Hüftprothesenwechsel analysiert und erlösrelevante Einflussfaktoren im aG-DRG-System dargestellt werden. Eine spezifische Fragestellung war, ob das aG-DRG-System finanzielle Unterschiede hinsichtlich des Fast- vs. Slow-Track-Managements von periprothetischen Hüftinfektionen bewirkt und dadurch gewisse finanzielle Anreize zur Bevorzugung einer Methode geschaffen werden könnten.

## Methodik

### Fallsimulation

In Form einer Fallsimulation wurden die Gesamterlöse eines zweizeitigen septischen Hüftprothesenwechsels sowohl mit kurzem Intervall (Fast-Track) als auch mit langem Intervall (Slow-Track) ermittelt.

Mithilfe der Grouper-Software 3M KODIP® Suite (Version 2020; 3M Deutschland, Neuss, Deutschland) wurden für Fast-Track und Slow-Track jeweils zwei verschiedene Fallbeispiele einer chronischen periprothetischen Hüftinfektion mit Nachweis eines multisensiblen Staphylococcus aureus (MSSA) simuliert. Beim Fast-Track erfolgte der zweizeitige Prothesenwechsel in einem stationären Aufenthalt mit kurzem Intervall, beim Slow-Track wurde der Prothesenwechsel mit langem Intervall in zwei stationären Aufenthalten durchgeführt.

Die operativen Eingriffe des Hüftprothesenwechsels umfassten im ersten Schritt den Prothesenausbau mit Implantation eines Spacers aus antibiotikahaltigem Knochenzement sowie die Reimplantation einer zementfreien Hüftprothese nach Spacerentfernung im zweiten Schritt und wurden anhand der entsprechenden Operationen- und Prozedurenschlüssel (OPS) kodiert.

### Simulierte Fallbeispiele

#### Fast-Track

Im Fallbeispiel 1a ohne weitere Nebendiagnosen (Tab. 1) betrug die stationäre Verweildauer 25 Tage mit kurzem prothesenfreien Intervall von 2 Wochen. Im Fallbeispiel 1b (Tab. 2) wurden zusätzlich Nebendiagnosen kodiert, die einen Einfluss auf den PCCL (Patient Clinical Complexity Level) haben können, in der Annahme, dass dies zu einem höheren DRG-Erlös führt. Die Verweildauer war 42 Tage bei einem Interimsintervall von 3 Wochen. In beiden Fällen wurde nur mit einem stationären Aufenthalt kalkuliert.

**Table 1:** 

*Diagnosen (ICD-10-GM)*		
Hauptdiagnose	M00.05 R	Arthritis und Polyarthritis durch Staphylokokken Beckenregion und Oberschenkel (Becken, Femur, Gesäß, Hüfte, Hüftgelenk, Iliosakralgelenk)
Nebendiagnosen	B95.6!	Staphylococcus aureus als Ursache von Krankheiten, die in anderen Kapiteln klassifiziert sind
	T84.5 R	Infektion und entzündliche Reaktion durch eine Gelenkendoprothese
	Z96.64 R	Vorhandensein einer Hüftgelenkprothese
*Prozeduren (OPS)*		
5–821.7 R	01.04.2020	Entfernung einer Totalendoprothese am Hüftgelenk
5‑780.6f R	01.04.2020	Inzision am Knochen, septisch und aseptisch: Debridement Femur proximal
5‑780.6d	01.04.2020	Inzision am Knochen, septisch und aseptisch: Debridement Becken
5‑829.9	01.04.2020	Einbringen von Abstandshaltern (z. B. nach Entfernung einer Endoprothese)
5‑785.1f R	01.04.2020	Implantation von Knochenzement mit Antibiotikumzusatz: Femur proximal
5‑829.g	15.04.2020	Entfernung von Abstandshaltern
5–820.00 R	15.04.2020	Implantation einer Endoprothese am Hüftgelenk: Totalendoprothese nichtzementiert
5‑829.*n*	15.04.2020	Implantation einer Endoprothese nach vorheriger Explantation
*Grouper-Ergebnis*		
DRG: I03A		Revision oder Ersatz des Hüftgelenkes mit kompl. Diagnose od. Arthrodese od. Alter < 16 Jahre oder beidseitige od. mehrere gr. Eingr. an Gelenken der unt. Extr. mit kompl. Eingriff, mit äuß. schw. CC oder mehrzeitigem Wechsel oder Eingr. an mehr. Lok.
MDC: 08 PCCL: 3 Partition: O		Krankheiten und Störungen an Muskel-Skelett-System und Bindegewebe
VWD: 25		Verwendete Verweildauer
Rg: 5,2130 Basisfallwert: 3660,92 €		Relativgewicht
DRG-Erlös: 19.084,38 €		DRG-Erlös ohne Zu‑/Abschlag
Zu‑/Abschlag: 0,00 € ZE-Betrag: 0,00 €		VWD = 25, UGVD 1. Tag Ab. = 10, mVD = 32,6, OGVD 1. Tag Zu. = 51
Pflegeerlös: 4880,25 €		Erlös aus der Pflege (ab 2020)
Gesamterlös: 23.964,63 €		Summe aus DRG-Erlös, Zu- und Abschlägen, Zusatzentgelte und Pflege

*CC* Komplikationen oder Komorbiditäten, *DRG* Diagnosis Related Groups, *MDC* „Major Diagnostic Category“, *mVD* mittlere Verweildauer, *OGVD* obere Grenzverweildauer, *OPS* Operationen- und Prozedurenschlüssel, *PCCL* Patient Clinical Complexity Level, *UGVD* untere Grenzverweildauer, *VWD* Verweildauer, *ZE* Zusatzentgelt

**Table 2:** 

*Diagnosen (ICD-10-GM)*		
Hauptdiagnose	M00.05 R	Arthritis und Polyarthritis durch Staphylokokken Beckenregion und Oberschenkel (Becken, Femur, Gesäß, Hüfte, Hüftgelenk, Iliosakralgelenk)
Nebendiagnosen	B95.6!	Staphylococcus aureus als Ursache von Krankheiten, die in anderen Kapiteln klassifiziert sind
	T84.5 R	Infektion und entzündliche Reaktion durch eine Gelenkendoprothese
	Z96.64 R	Vorhandensein einer Hüftgelenkprothese
	D62	Akute Blutungsanämie
	N18.3	Chronische Nierenkrankheit, Stadium 3
	E87.6	Hypokaliämie
	J90 B	Pleuraerguss, andernorts nicht klassifiziert
	E66.02	Adipositas Grad III (WHO) durch übermäßige Kalorienzufuhr bei Patienten von 18 Jahren und älter
	L89.27 L	Dekubitus 3. Grades, Ferse
	I80.28 R	Thrombose, Phlebitis und Thrombophlebitis sonstiger tiefer Gefäße der unteren Extremitäten
*Prozeduren (OPS)*		
5–821.7 R	01.04.2020	Entfernung einer Totalendoprothese am Hüftgelenk
5‑780.6f R	01.04.2020	Inzision am Knochen, septisch und aseptisch: Debridement Femur proximal
5‑780.6d	01.04.2020	Inzision am Knochen, septisch und aseptisch: Debridement Becken
5‑829.9	01.04.2020	Einbringen von Abstandshaltern (z. B. nach Entfernung einer Endoprothese)
5‑785.1f R	01.04.2020	Implantation von Knochenzement mit Antibiotikumzusatz: Femur proximal
5‑829.g	22.04.2020	Entfernung von Abstandshaltern
5–820.00 R	22.04.2020	Implantation einer Endoprothese am Hüftgelenk: Totalendoprothese nichtzementiert
5‑829.n	22.04.2020	Implantation einer Endoprothese nach vorheriger Explantation
8‑800.c0	02.04.2020	Transfusion von Erythrozytenkonzentrat: 1 TE bis unter 6 TE
*Grouper-Ergebnis*		
DRG: I03A		Revision oder Ersatz des Hüftgelenkes mit kompl. Diagnose od. Arthrodese od. Alter < 16 Jahre oder beidseitige od. mehrere gr. Eingr. an Gelenken der unt. Extr. mit kompl. Eingriff, mit äuß. schw. CC oder mehrzeitigem Wechsel oder Eingr. an mehr. Lok.
MDC: 08 PCCL: 4 Partition: O		Krankheiten und Störungen an Muskel-Skelett-System und Bindegewebe
VWD: 42		Verwendete Verweildauer
Rg: 5,2130 Basisfallwert: 3660,92 €		Relativgewicht
DRG-Erlös: 19.084,38 €		DRG-Erlös ohne Zu‑/Abschlag
Zu‑/Abschlag: 0,00 € ZE-Betrag: 0,00 €		VWD = 42, UGVD 1. Tag Ab. = 10, mVD = 32,6, OGVD 1. Tag Zu. = 51
Pflegeerlös: 8198,82 €		Erlös aus der Pflege (ab 2020)
Gesamterlös: 27.283,20 €		Summe aus DRG-Erlös, Zu- und Abschlägen, Zusatzentgelte und Pflege

*CC* Komplikationen oder Komorbiditäten, *DRG* Diagnosis Related Groups, *MDC* „Major Diagnostic Category“, *mVD* mittlere Verweildauer, *OGVD* obere Grenzverweildauer, *OPS* Operationen- und Prozedurenschlüssel, *PCCL* Patient Clinical Complexity Level, *TE* Transfusionseinheit, *UGVD* untere Grenzverweildauer, *VWD* Verweildauer, *ZE* Zusatzentgelt

#### Slow-Track

Beim Slow-Track wurden pro Fallbeispiel zwei stationäre Aufenthalte simuliert, wobei im ersten Aufenthalt der Hüftprothesenausbau erfolgte und mit einem langen Intervall im zweiten Aufenthalt die Reimplantation der Hüftprothese durchgeführt wurde. Die Verweildauer und Erlöse beider stationärer Aufenthalte wurden jeweils addiert.

Im Fallbeispiel 2a (Tab. 3) wurde ein Behandlungsfall simuliert, bei dem in beiden stationären Aufenthalten keine weiteren Nebendiagnosen vorlagen, die Gesamtverweildauer 25 Tage betrug und der Wiedereinbau der Prothese nach 6 Wochen durchgeführt wurde. Um einen möglichen Einfluss auf den Gesamterlös zu ermitteln, wurden im Fallbeispiel 2b (Tab. 4) zusätzliche Nebendiagnosen in beiden stationären Aufenthalten kodiert und die Gesamtverweildauer auf 42 Tage verlängert. Die Reimplantation der Prothese erfolgte nach 8 Wochen.

**Table 3:** 

*Diagnosen (ICD-10-GM)*		*1. Stationärer Behandlungsfall*	*Diagnosen (ICD-10-GM)*		*2. Stationärer Behandlungsfall*
HD:	M00.05 R	Arthritis und Polyarthritis durch Staphylokokken Beckenregion und Oberschenkel (Becken, Femur, Gesäß, Hüfte, Hüftgelenk, Iliosakralgelenk)	HD:	M00.05 R	Arthritis und Polyarthritis durch Staphylokokken Beckenregion und Oberschenkel (Becken, Femur, Gesäß, Hüfte, Hüftgelenk, Iliosakralgelenk)
ND:	B95.6!	Staphylococcus aureus als Ursache von Krankheiten, die in anderen Kapiteln klassifiziert sind	ND:	B95.6!	Staphylococcus aureus als Ursache von Krankheiten, die in anderen Kapiteln klassifiziert sind
	T84.5 R	Infektion und entzündliche Reaktion durch eine Gelenkendoprothese			
	Z96.64 R	Vorhandensein einer Hüftgelenkprothese			
*Prozeduren (OPS)*			*Prozeduren (OPS)*		
5–821.7 R	01.04.2020	Entfernung einer Totalendoprothese am Hüftgelenk	5‑829.g	13.05.2020	Entfernung von Abstandshaltern
5‑780.6f R	01.04.2020	Inzision am Knochen, septisch und aseptisch: Debridement Femur proximal			
5‑780.6d	01.04.2020	Inzision am Knochen, septisch und aseptisch: Debridement Becken	5–820.00 R	13.05.2020	Implantation einer Endoprothese am Hüftgelenk: Totalendoprothese nichtzementiert
5‑829.9	01.04.2020	Einbringen von Abstandshaltern (z. B. nach Entfernung einer Endoprothese)			
5‑785.1f R	01.04.2020	Implantation von Knochenzement mit Antibiotikumzusatz: Femur proximal	5‑829.n	13.05.2020	Implantation einer Endoprothese nach vorheriger Explantation
*Grouper-Ergebnis*			*Grouper-Ergebnis*		
DRG: I08C		And. Eingr. Hüftgel. mit mäßig kompl. Eingriff ohne best. kompl. Faktoren, ohne best. kompl. Proz. od. m. kompl. Proz. od. Diagn. od. Alter < 6 J. od. Eingr. in Komb. Hüftgel. und ob. Extr. od. WS od. m. offener Rep. Beckenringfraktur od. m. Komplexbeh.	DRG: I04Z		Implantation, Wechsel oder Entfernung einer Endoprothese am Kniegelenk mit komplizierender Diagnose oder Arthrodese oder Implantation einer Endoprothese nach vorheriger Explantation oder periprothetische Fraktur an der Schulter oder am Knie
MDC: 08 PCCL: 2 Partition: O		Krankheiten und Störungen an Muskel-Skelett-System und Bindegewebe	MDC: 08 PCCL: 0 Partition: O		Krankheiten und Störungen an Muskel-Skelett-System und Bindegewebe
VWD: 15		Verwendete Verweildauer	VWD: 10		Verwendete Verweildauer
Rg: 3,1030 Basisfallwert: 3660,92 €		Relativgewicht	Rg: 3,2020 Basisfallwert: 3660,92 €		Relativgewicht
DRG-Erlös: 11.359,83 €		DRG-Erlös ohne Zu‑/Abschlag	DRG-Erlös: 11.722,27 €		DRG-Erlös ohne Zu‑/Abschlag
Zu‑/Abschlag: 0,00 € ZE-Betrag: 0,00 €		VWD = 15, UGVD 1. Tag Ab. = 5, mVD = 17,8, OGVD 1. Tag Zu. = 33	Zu‑/Abschlag: 0,00 € ZE-Betrag: 0,00 €		VWD = 10, UGVD 1. Tag Ab. = 5, mVD = 16,7, OGVD 1. Tag Zu. = 30
Pflegeerlös: 2895,15 €		Erlös aus der Pflege (ab 2020)	Pflegeerlös: 1574,20 €		Erlös aus der Pflege (ab 2020)
Gesamterlös: 14.254,98 €		Summe aus DRG-Erlös, Zu- und Abschlägen, Zusatzentgelte und Pflege	Gesamterlös: 13.296,47 €		Summe aus DRG-Erlös, Zu- und Abschlägen, Zusatzentgelte und Pflege
*Summe aus beiden stationären Behandlungsfällen*					
VWD: 25		Verwendete Verweildauer			
DRG-Erlös: 23.082,10 €		DRG-Erlös ohne Zu‑/Abschlag			
Pflegeerlös: 4469,35 €		Erlös aus der Pflege (ab 2020)			
Gesamterlös: 27.551,45 €		Summe aus DRG-Erlös, Zu- und Abschlägen, Zusatzentgelte und Pflege			

*DRG* Diagnosis Related Groups, *MDC* „Major Diagnostic Category“, *mVD* mittlere Verweildauer, *OGVD* obere Grenzverweildauer, *OPS* Operationen- und Prozedurenschlüssel, *PCCL* Patient Clinical Complexity Level, *UGVD* untere Grenzverweildauer, *VWD* Verweildauer, *WS* Wirbelsäule, ZE Zusatzentgelt

**Table 4:** 

*Diagnosen (ICD-10-GM)*		*1. Stationärer Behandlungsfall*	*Diagnosen (ICD-10-GM)*		*2. Stationärer Behandlungsfall*
HD:	M00.05 R	Arthritis und Polyarthritis durch Staphylokokken Beckenregion und Oberschenkel (Becken, Femur, Gesäß, Hüfte, Hüftgelenk, Iliosakralgelenk)	HD:	M00.05 R	Arthritis und Polyarthritis durch Staphylokokken Beckenregion und Oberschenkel (Becken, Femur, Gesäß, Hüfte, Hüftgelenk, Iliosakralgelenk)
ND:	B95.6!	Staphylococcus aureus als Ursache von Krankheiten, die in anderen Kapiteln klassifiziert sind	ND:	B95.6!	Staphylococcus aureus als Ursache von Krankheiten, die in anderen Kapiteln klassifiziert sind
	T84.5 R	Infektion und entzündliche Reaktion durch eine Gelenkendoprothese			
	Z96.64 R	Vorhandensein einer Hüftgelenkprothese		D62	Akute Blutungsanämie
	N18.3	Chronische Nierenkrankheit, Stadium 3		E66.02	Adipositas Grad III (WHO) durch übermäßige Kalorienzufuhr bei Patienten von 18 Jahren und älter
	E87.6	Hypokaliämie		J90 B	Pleuraerguss, andernorts nicht klassifiziert
	L89.27 L	Dekubitus 3. Grades, Ferse		I80.28 R	Thrombose, Phlebitis und Thrombophlebitis sonstiger tiefer Gefäße der unteren Extremitäten
	D62	Akute Blutungsanämie		L89.27 L	Dekubitus 3. Grades, Ferse
*Prozeduren (OPS)*			*Prozeduren (OPS)*		
5–821.7 R	01.04.2020	Entfernung einer Totalendoprothese am Hüftgelenk	5‑829.g	27.05.2020	Entfernung von Abstandshaltern
5‑780.6f R	01.04.2020	Inzision am Knochen, septisch und aseptisch: Debridement Femur proximal	5–820.00 R	27.05.2020	Implantation einer Endoprothese am Hüftgelenk: Totalendoprothese nichtzementiert
5‑780.6d	01.04.2020	Inzision am Knochen, septisch und aseptisch: Debridement Becken			
5‑829.9	01.04.2020	Einbringen von Abstandshaltern (z. B. nach Entfernung einer Endoprothese)			
5‑785.1f R	01.04.2020	Implantation von Knochenzement mit Antibiotikumzusatz: Femur proximal	5‑829.n	27.05.2020	Implantation einer Endoprothese nach vorheriger Explantation
8‑800.c0	02.04.2020	Transfusion von Erythrozytenkonzentrat: 1 TE bis unter 6 TE	8‑800.c0	28.05.2020	Transfusion von Erythrozytenkonzentrat: 1 TE bis unter 6 TE
*Grouper-Ergebnis*			*Grouper-Ergebnis*		
DRG: I08B		And. Eingr. Hüftgel. mit kompl. Proz. od. Eingr. in Komb. Hüftg. und ob. Extr. od. WS od. best. kompl. Fakt., oh. best. Eingriffe mit best. Diag., oh. best. Beckenrepos., oh. kompl. Fakt. od. kompl. Proz. od. Diag. od. äuß. schw. CC oh. BNB WS und Becken	DRG: I03A		Revision oder Ersatz des Hüftgelenkes mit kompl. Diagnose od. Arthrodese od. Alter < 16 Jahre oder beidseitige od. mehrere gr. Eingr. an Gelenken der unt. Extr. mit kompl. Eingriff, mit äuß. schw. CC oder mehrzeitigem Wechsel oder Eingr. an mehr. Lok.
MDC: 08 PCCL: 4 Partition: O		Krankheiten und Störungen an Muskel-Skelett-System und Bindegewebe	MDC: 08 PCCL: 4 Partition: O		Krankheiten und Störungen an Muskel-Skelett-System und Bindegewebe
VWD: 21		Verwendete Verweildauer	VWD: 21		Verwendete Verweildauer
Rg: 3,6260 Basisfallwert: 3660,92 €		Relativgewicht	Rg: 5,2130 Basisfallwert: 3660,92 €		Relativgewicht
DRG-Erlös: 13.274,50 €		DRG-Erlös ohne Zu‑/Abschlag	DRG-Erlös: 19.084,38 €		DRG-Erlös ohne Zu‑/Abschlag
Zu‑/Abschlag: 0,00 € ZE-Betrag: 0,00 €		VWD = 21, UGVD 1. Tag Ab. = 6, mVD = 20,8, OGVD 1. Tag Zu. = 39	Zu‑/Abschlag: 0,00 € ZE-Betrag: 0,00 €		VWD = 21, UGVD 1. Tag Ab. = 10, mVD = 32,6, OGVD 1. Tag Zu. = 51
Pflegeerlös: 4240,53 €		Erlös aus der Pflege (ab 2020)	Pflegeerlös: 4099,41 €		Erlös aus der Pflege (ab 2020)
Gesamterlös: 17.515,03 €		Summe aus DRG-Erlös, Zu- und Abschlägen, Zusatzentgelte und Pflege	Gesamterlös: 23.183,79 €		Summe aus DRG-Erlös, Zu- und Abschlägen, Zusatzentgelte und Pflege
*Summe aus beiden stationären Behandlungsfällen*					
VWD: 42		Verwendete Verweildauer			
DRG-Erlös: 32.358,88 €		DRG-Erlös ohne Zu‑/Abschlag			
Pflegeerlös: 8339,94 €		Erlös aus der Pflege (ab 2020)			
Gesamterlös: 40.698,82 €		Summe aus DRG-Erlös, Zu- und Abschlägen, Zusatzentgelte und Pflege			

*CC* Komplikationen oder Komorbiditäten, *DRG* Diagnosis Related Groups, *MDC* „Major Diagnostic Category“, mVD mittlere Verweildauer, *OGVD* obere Grenzverweildauer, *OPS* Operationen- und Prozedurenschlüssel, *PCCL* Patient Clinical Complexity Level, *TE* Transfusionseinheit, *UGVD* untere Grenzverweildauer, *VWD* Verweildauer, *WS* Wirbelsäule, *ZE* Zusatzentgelt

### Auswertung der Fallsimulationen

Die Gesamterlöse wurden aus dem DRG-Erlös der ermittelten DRG (I03A, I08B, I08C und I04Z) bei einem Landesbasisfallwert von 3660,92 € (LBFW 2020 Bayern) und dem Pflegeerlös (tagesbezogener Pflegeentgeltwert 185 €) berechnet. Zusatzentgelte und Verweildauerzuschläge blieben in der Fallsimulation unberücksichtigt.

Zur Ermittlung der Erlösdifferenz ∆ wurden die Gesamterlöse der Fallbeispiele mit Fast-Track und Slow-Track jeweils miteinander verglichen (Tab. 5).

**Table 5:** 

	Ohne ND, VWD 25 Tage	Mit ND, VWD 42 Tage	∆
Fast-Track	23.965 €	27.283 €	3318 €
Slow-Track	27.551 €	40.699 €	13.148 €
∆	3586 €	13.416 €	–

### Reale Patientenfälle

Um zusätzlich die reale Erlössituation im aG-DRG-System aufzuzeigen, wurden zwei Behandlungsfälle des Jahres 2020 mit zweizeitigem septischem Hüftprothesenwechsel mit Fast-Track und Slow-Track, die in der Klinik und Poliklinik für Unfallchirurgie des Universitätsklinikums Regensburg behandelt wurden, hinsichtlich der Gesamterlöse miteinander verglichen und die Erlösdifferenz ∆ berechnet. Die Kodierung der Diagnosen (ICD-10-GM) und Prozeduren (OPS) sowie die Eingruppierung der Behandlungsfälle wurden mit der Grouper-Software 3M KODIP Suite (Version 2020) durchgeführt.

#### Patientenfall 1 (Tab. 6)

Es handelte sich um eine 68-jährige immobile Patientin mit periprothetischer Hüftinfektion links nach auswärtiger Erstimplantation einer zementfreien Hüftprothese im Juli 2020, bei der ein zweizeitiger Prothesenwechsel als Fast-Track mit kurzem Intervall von 3 Wochen in einem stationären Aufenthalt vom 17.09.2020 bis 19.10.2020 durchgeführt wurde. Am 17.09.2020 erfolgte der Hüft-TEP-Ausbau und die Implantation eines antibiotikahaltigen Spacers. Histologisch konnte eine Osteomyelitis im Bereich des proximalen Femurs gesichert werden bei mikrobiologischem Nachweis eines Staphylococcus epidermidis und Cutibacterium acnes. Am 09.10.2020 erfolgte der Wiedereinbau einer zementierten Hüftprothese. Die stationäre Verweildauer betrug 32 Tage. An Komorbiditäten waren eine arterielle Hypertonie, eine Hypothyreose sowie eine Adipositas Grad II bekannt. Postoperativ wurde nach dem zweiten Eingriff eine tiefe Beinvenenthrombose links diagnostiziert und therapiert. Zudem erfolgte eine Kaliumsubstitution und die Transfusion eines Erythrozytenkonzentrates.

**Table 6:** 

*Diagnosen (ICD-10-GM)*		*Stationärer Behandlungsfall (17.09.2020–19.10.2020)*
Hauptdiagnose	M86.65 L	Sonstige chronische Osteomyelitis: Beckenregion und Oberschenkel (Becken, Femur, Gesäß, Hüfte, Hüftgelenk, Iliosakralgelenk)
Nebendiagnosen	B95.7!	Sonstige Staphylokokken als Ursache von Krankheiten, die in anderen Kapiteln klassifiziert sind
	B95.91!	Sonstige näher bezeichnete grampositive anaerobe, nicht sporenbildende Erreger als Ursache von Krankheiten, die in anderen Kapiteln klassifiziert sind
	T84.5 L	Infektion und entzündliche Reaktion durch eine Gelenkendoprothese
	Z96.64 L	Vorhandensein einer Hüftgelenkprothese
	I10.00	Benigne essenzielle Hypertonie ohne Angabe einer hypertensiven Krise
	E66.01	Adipositas Grad II (WHO) durch übermäßige Kalorienzufuhr bei Patienten von 18 Jahren und älter
	E03.9	Hypothyreose, nicht näher bezeichnet
	D62	Akute Blutungsanämie
	E87.6	Hypokaliämie
	I80.28 L	Thrombose, Phlebitis und Thrombophlebitis sonstiger tiefer Gefäße der unteren Extremitäten
	R26.3	Immobilität
	U50.40	Schwere motorische Funktionseinschränkung, Barthel-Index 20–35 Punkte
	U51.00	Keine oder leichte kognitive Funktionseinschränkung, erweiterter Barthel-Index 70–90 Punkte
	Z50.1!	Sonstige Physiotherapie
	Z46.7	Versorgen mit und Anpassen eines orthopädischen Hilfsmittels
	Z11	Spezielle Verfahren zur Untersuchung auf infektiöse und parasitäre Krankheiten
	U99.0!	Spezielle Verfahren zur Untersuchung auf SARS-CoV‑2
*Prozeduren (OPS)*		
5–821.7 L	17.09.2020	Entfernung einer Totalendoprothese am Hüftgelenk
5‑780.6f L	17.09.2020	Inzision am Knochen, septisch und aseptisch: Debridement Femur proximal
5‑780.6d	17.09.2020	Inzision am Knochen, septisch und aseptisch: Debridement Becken
5‑829.9	17.09.2020	Einbringen von Abstandshaltern (z. B. nach Entfernung einer Endoprothese)
5‑785.1f L	17.09.2020	Implantation von Knochenzement mit Antibiotikumzusatz: Femur proximal
5‑829.g	09.10.2020	Entfernung von Abstandshaltern
5–820.01 L	09.10.2020	Implantation einer Endoprothese am Hüftgelenk: Totalendoprothese zementiert
5‑829.n	09.10.2020	Implantation einer Endoprothese nach vorheriger Explantation
8‑800.c0	19.09.2020	Transfusion von Erythrozytenkonzentrat: 1 TE bis unter 6 TE
8‑831.0	17.09.2020	Legen eines Katheters in zentralvenöse Gefäße
8‑831.0	09.10.2020	Legen eines Katheters in zentralvenöse Gefäße
9‑401.01	21.09.2020	Sozialrechtliche Beratung, mehr als 2 h bis 4 h
*Grouper-Ergebnis*		
DRG: I03A		Revision oder Ersatz des Hüftgelenkes mit kompl. Diagnose od. Arthrodese od. Alter < 16 Jahre oder beidseitige od. mehrere gr. Eingr. an Gelenken der unt. Extr. mit kompl. Eingriff, mit äuß. schw. CC oder mehrzeitigem Wechsel oder Eingr. an mehr. Lok.
MDC: 08 PCCL: 4 Partition: O		Krankheiten und Störungen an Muskel-Skelett-System und Bindegewebe
VWD: 32		Verwendete Verweildauer
Rg: 5,2130 Basisfallwert: 3660,92 €		Relativgewicht
DRG-Erlös: 19.084,38 €		DRG-Erlös ohne Zu‑/Abschlag
Zu‑/Abschlag: 0,00 €		VWD = 32, UGVD 1. Tag Ab. = 10, mVD = 32,6, OGVD 1. Tag Zu. = 51
ZE-Betrag: 52,50 €		Zusatzentgelt für Corona-Test
Pflegeerlös: 6246,72 €		Erlös aus der Pflege (ab 2020)
Gesamterlös: 25.383,60 €		Summe aus DRG-Erlös, Zu- und Abschlägen, Zusatzentgelte und Pflege

*CC* Komplikationen oder Komorbiditäten, *DRG* Diagnosis Related Groups, *MDC* „Major Diagnostic Category“, mVD mittlere Verweildauer, *OGVD* obere Grenzverweildauer, *OPS* Operationen- und Prozedurenschlüssel, *PCCL* Patient Clinical Complexity Level, *SARS-CoV 2* „severe acute respiratory syndrome coronavirus type 2“, *TE* Transfusionseinheit, *UGVD* untere Grenzverweildauer, *VWD* Verweildauer, *ZE* Zusatzentgelt

#### Patientenfall 2 (Tab. 7)

Bei der 55-jährigen Patientin bestand eine periprothetische Reinfektion der linken Hüfte mit Nachweis eines Staphylococcus epidermidis bei Zustand nach Reimplantation einer zementfreien Hüftprothese und Cerclagenosteosynthese am proximalen Femur links im März 2020. Es erfolgte ein zweizeitiger Prothesenwechsel mit langem Intervall von 11 Wochen (Slow-Track) in zwei stationären Aufenthalten vom 18.09.2020 bis 09.10.2020 sowie vom 01.12.2020 bis 10.12.2020, wobei die Gesamtverweildauer bei 30 Tagen lag. Am 18.09.2020 wurde der Ausbau der Hüftprothese und die Implantation eines antibiotikahaltigen Spacers durchgeführt. Die Histologie zeigte eine Osteomyelitis im Bereich des proximalen Femurs. Nach 21 Tagen wurde die Patientin entlassen und am 01.12.2020 erneut stationär aufgenommen. Es erfolgte am 02.12.2020 die Implantation einer zementfreien silberbeschichteten modularen Hüftprothese und am 10.12.2020 die Entlassung nach insgesamt 9 Tagen. An Nebendiagnosen lagen eine arterielle Hypertonie, eine multiple Sklerose, eine Adipositas Grad III, eine postoperative Anämie, eine Hyponatriämie und eine Hypokaliämie vor, die jeweils einen entsprechenden Mehraufwand erforderten.

**Table 7:** 

*Diagnosen (ICD-10-GM)*		*1. Stationärer Behandlungsfall (18.09.2020–09.10.2020)*	*Diagnosen (ICD-10-GM)*		*2. Stationärer Behandlungsfall (01.12.2020–10.12.2020)*
HD:	M86.65 L	Sonstige chronische Osteomyelitis: Beckenregion und Oberschenkel (Becken, Femur, Gesäß, Hüfte, Hüftgelenk, Iliosakralgelenk)	HD:	M86.65 L	Sonstige chronische Osteomyelitis: Beckenregion und Oberschenkel (Becken, Femur, Gesäß, Hüfte, Hüftgelenk, Iliosakralgelenk)
ND:	B95.7!	Sonstige Staphylokokken als Ursache von Krankheiten, die in anderen Kapiteln klassifiziert sind	ND:	B95.7!	Sonstige Staphylokokken als Ursache von Krankheiten, die in anderen Kapiteln klassifiziert sind
	T84.5 L	Infektion und entzündliche Reaktion durch eine Gelenkendoprothese		I10.00	Benigne essenzielle Hypertonie ohne Angabe einer hypertensiven Krise
	Z96.64 L	Vorhandensein einer Hüftgelenkprothese		G35.20	Multiple Sklerose (Encephalomyelitis disseminata) mit primär-chronischem Verlauf, ohne Angabe einer akuten Exazerbation oder Progression
	I10.00	Benigne essenzielle Hypertonie ohne Angabe einer hypertensiven Krise		E66.02	Adipositas Grad III (WHO) durch übermäßige Kalorienzufuhr bei Patienten von 18 Jahren und älter
	G35.20	Multiple Sklerose (Encephalomyelitis disseminata) mit primär-chronischem Verlauf, ohne Angabe einer akuten Exazerbation oder Progression		D62	Akute Blutungsanämie
	E66.02	Adipositas Grad III (WHO) durch übermäßige Kalorienzufuhr bei Patienten von 18 Jahren und älter		E87.6	Hypokaliämie
	D62	Akute Blutungsanämie		Z47.0	Entfernung einer Metallplatte oder einer anderen inneren Fixationsvorrichtung
	E87.1	Hypoosmolalität und Hyponatriämie		U50.30	Mittelschwere motorische Funktionseinschränkung, Barthel-Index 40–55 Punkte
	Z47.0	Entfernung einer Metallplatte oder einer anderen inneren Fixationsvorrichtung		U51.00	Keine oder leichte kognitive Funktionseinschränkung, erweiterter Barthel-Index 70–90 Punkte
	U50.20	Mittlere motorische Funktionseinschränkung, Barthel-Index 60–75 Punkte		Z50.1!	Sonstige Physiotherapie
	U51.00	Keine oder leichte kognitive Funktionseinschränkung, erweiterter Barthel-Index 70–90 Punkte		Z98.8	Sonstige näher bezeichnete Zustände nach chirurgischen Eingriffen
	Z50.1!	Sonstige Physiotherapie		Z11 U99.0! (siehe links, 1. stat. Behandlungsfall)	Spezielle Verfahren zur Untersuchung auf infektiöse und parasitäre Krankheiten Spezielle Verfahren zur Untersuchung auf SARS-CoV-2 (siehe links, 1. stat. Behandlungsfall)
	Z11	Spezielle Verfahren zur Untersuchung auf infektiöse und parasitäre Krankheiten			
	U99.0!	Spezielle Verfahren zur Untersuchung auf SARS-CoV‑2			
*Prozeduren (OPS)*			*Prozeduren (OPS)*		
5–821.7 L	18.09.2020	Entfernung einer Totalendoprothese am Hüftgelenk	5‑829.g	02.12.2020	Entfernung von Abstandshaltern
5‑787.2f L	18.09.2020	Entfernung einer Zuggurtung/Cerclage: Femur proximal	5‑787.2f L	02.12.2020	Entfernung einer Zuggurtung/Cerclage: Femur proximal
5‑780.6f L	18.09.2020	Inzision am Knochen, septisch und aseptisch: Debridement Femur proximal	5–820.20 L	02.12.2020	Implantation einer Endoprothese am Hüftgelenk: Totalendoprothese, Sonderprothese nichtzementiert
5‑780.6d	18.09.2020	Inzision am Knochen, septisch und aseptisch: Debridement Becken	5‑829.n	02.12.2020	Implantation einer Endoprothese nach vorheriger Explantation
5‑781.af L	18.09.2020	Osteotomie ohne Achskorrektur: Femur proximal	5‑829.k2	02.12.2020	Implantation einer modularen Endoprothese oder (Teil‑)Wechsel in eine modulare Endoprothese bei knöcherner Defektsituation und ggf. Knochen(teil)ersatz, Schaftkomponente mit einer dem Knochendefekt entsprechenden Länge und Dicke
5‑829.9	18.09.2020	Einbringen von Abstandshaltern (z. B. nach Entfernung einer Endoprothese)	5‑829.jx	02.12.2020	Verwendung von beschichteten Endoprothesen mit Silberbeschichtung
5‑785.1f L	18.09.2020	Implantation von Knochenzement mit Antibiotikumzusatz: Femur proximal	5‑786.1	02.12.2020	Osteosyntheseverfahren durch Draht oder Zuggurtung/Cerclage
5‑786.1	18.09.2020	Osteosyntheseverfahren durch Draht oder Zuggurtung/Cerclage	8‑800.c0	03.12.2020	Transfusion von Erythrozytenkonzentrat: 1 TE bis unter 6 TE
8‑800.c0	18.09.2020	Transfusion von Erythrozytenkonzentrat: 1 TE bis unter 6 TE	8‑930	02.12.2020	Monitoring von Atmung, Herz und Kreislauf ohne Messung des Pulmonalarteriendruckes und des zentralen Venendruckes
8‑831.0	18.09.2020	Legen eines Katheters in zentralvenöse Gefäße	9‑401.01	04.12.2020	Sozialrechtliche Beratung, mehr als 2 h bis 4 h
8‑831.2	21.09.2020	Wechsel eines zentralvenösen Katheters	9‑984.b	04.12.2020	Erfolgter Antrag auf Einstufung in einen Pflegegrad
9‑401.01	24.09.2020	Sozialrechtliche Beratung, mehr als 2 h bis 4 h			
*Grouper-Ergebnis*		*1. Stationärer Behandlungsfall (18.09.2020–09.10.2020)*	*Grouper-Ergebnis*		*2. Stationärer Behandlungsfall (01.12.2020–10.12.2020)*
DRG: I03A		Revision oder Ersatz des Hüftgelenkes mit kompl. Diagnose od. Arthrodese od. Alter < 16 Jahre oder beidseitige od. mehrere gr. Eingr. an Gelenken der unt. Extr. mit kompl. Eingriff, mit äuß. schw. CC oder mehrzeitigem Wechsel oder Eingr. an mehr. Lok.	DRG: I04Z		Implantation, Wechsel oder Entfernung einer Endoprothese am Kniegelenk mit komplizierender Diagnose oder Arthrodese oder Implantation einer Endoprothese nach vorheriger Explantation oder periprothetische Fraktur an der Schulter oder am Knie
MDC: 08 PCCL: 4 Partition: O		Krankheiten und Störungen an Muskel-Skelett-System und Bindegewebe	MDC: 08 PCCL: 2 Partition: O		Krankheiten und Störungen an Muskel-Skelett-System und Bindegewebe
VWD: 21		Verwendete Verweildauer	VWD: 9		Verwendete Verweildauer
Rg: 5,2130 Basisfallwert: 3660,92 €		Relativgewicht	Rg: 3,2020 Basisfallwert: 3660,92 €		Relativgewicht
DRG-Erlös: 19.084,38 €		DRG-Erlös ohne Zu‑/Abschlag	DRG-Erlös: 11.722,27 €		DRG-Erlös ohne Zu‑/Abschlag
Zu‑/Abschlag: 0,00 €		VWD = 21, UGVD 1. Tag Ab. = 10, mVD = 32,6, OGVD 1. Tag Zu. = 51	Zu‑/Abschlag: 0,00 €		VWD = 9, UGVD 1. Tag Ab. = 5, mVD = 16,7, OGVD 1. Tag Zu. = 30
ZE-Betrag: 52,50 €		Zusatzentgelt für Corona-Test	ZE-Betrag: 1252,50 €		Zusatzentgelte für Corona-Test und modulare Prothese
Pflegeerlös: 4099,41 €		Erlös aus der Pflege (ab 2020)	Pflegeerlös: 1416,78 €		Erlös aus der Pflege (ab 2020)
Gesamterlös: 23.236,29 €		Summe aus DRG-Erlös, Zu- und Abschlägen, Zusatzentgelte und Pflege	Gesamterlös: 14.391,55 €		Summe aus DRG-Erlös, Zu- und Abschlägen, Zusatzentgelte und Pflege
*Summe aus beiden stationären Behandlungsfällen*					
VWD: 30		Verwendete Verweildauer			
DRG-Erlös: 30.806,65 €		DRG-Erlös ohne Zu‑/Abschlag			
Pflegeerlös: 5516,19 €		Erlös aus der Pflege (ab 2020)			
Gesamterlös: 37.627,84 €		Summe aus DRG-Erlös, Zu- und Abschlägen, Zusatzentgelte und Pflege			

*CC* Komplikationen oder Komorbiditäten, *DRG* Diagnosis Related Groups, *MDC* „Major Diagnostic Category“, mVD mittlere Verweildauer, *OGVD* obere Grenzverweildauer, *OPS* Operationen- und Prozedurenschlüssel, *PCCL* Patient Clinical Complexity Level, *SARS-CoV 2* „severe acute respiratory syndrome coronavirus type 2“, *TE* Transfusionseinheit, *UGVD* untere Grenzverweildauer, *VWD* Verweildauer, *ZE* Zusatzentgelt

### Auswertung der Patientenfälle

Die Gesamterlöse wurden aus dem DRG-Erlös der zugrundeliegenden DRG (I03A und I03A + I04Z) bei einem Landesbasisfallwert von 3660,92 € (LBFW 2020 Bayern), dem Pflegeerlös (tagesbezogener Pflegeentgeltwert 185 €) und den abrechenbaren Zusatzentgelten ermittelt und die Erlösdifferenz ∆ berechnet. Zu- oder Abschläge auf den DRG-Erlös ergaben sich bei beiden Fällen nicht.

## Ergebnisse

In den simulierten Fallbeispielen ergaben sich deutliche Erlösunterschiede für die Behandlungsalternativen.

### Fast-Track

Der Gesamterlös lag beim Fast-Track bei einer Verweildauer von 25 Tagen ohne weitere Nebendiagnosen bei 23.965 € (Tab. 1), bei einer Verweildauer von 42 Tagen mit zusätzlichen Nebendiagnosen bei 27.283 € (Tab. 2). Es zeigte sich, dass bei beiden Behandlungsfällen unabhängig von den Nebendiagnosen ein DRG-Erlös von 19.084 € (DRG I03A) erzielt wurde, sodass die im Fallbeispiel 1b kodierten CCL-relevanten Nebendiagnosen beim Fast-Track keinen Einfluss auf den DRG-Erlös hatten. Die Erlösdifferenz von 3318 € zwischen beiden Fallbeispielen mit Fast-Track (Tab. 5) resultierte bei unterschiedlicher Verweildauer aus dem tagesbezogenen Pflegeentgelt (25 Tage VWD: 4880 €, 42 Tage VWD: 8199 €).

### Slow-Track

Beim Slow-Track lag der Gesamterlös in beiden Fallbeispielen höher als beim Fast-Track, da hier zwei stationäre Behandlungsfälle abgerechnet werden konnten. Bei einer Verweildauer von 25 Tagen ohne weitere Nebendiagnosen wurde ein Gesamterlös von 27.551 € aus beiden stationären Fällen ermittelt (Tab. 3). Im Fallbeispiel 2b mit zusätzlichen Nebendiagnosen und 42 Tagen Verweildauer wurde ein deutlich höherer Gesamterlös von 40.699 € aus beiden stationären Fällen erzielt (Tab. 4). Die hohe Erlösdifferenz von 13.148 € zwischen beiden Fallbeispielen mit Slow-Track (Tab. 5) war zum einen auf die unterschiedliche Verweildauer mit dadurch differierendem Pflegeerlös zurückzuführen (25 Tage VWD: 4469 €, 42 Tage VWD: 8340 €). Zum anderen wirkten sich beim Slow-Track die kodierten Nebendiagnosen im Fallbeispiel 2b erlössteigernd aus und führten zu einem Anstieg des DRG-Erlöses um 9277 €. Der DRG-Erlös lag beim Slow-Track ohne Nebendiagnosen bei 23.082 € (DRG I08C + I04Z), beim Slow-Track mit Nebendiagnosen bei 32.359 € (DRG I08B + I03A).

Die Gegenüberstellung Fast-Track vs. Slow-Track ohne weitere Nebendiagnosen (VWD 25 Tage) zeigte eine Erlösdifferenz von 3586 € zum Vorteil des Slow-Tracks. Beim Vergleich Fast-Track vs. Slow-Track mit zusätzlichen Nebendiagnosen (VWD 42 Tage) war der Gesamterlös beim Slow-Track um 13.416 € höher als beim Fast-Track (Tab. 5).

Auch bei den realen Behandlungsfällen zeigte sich eine große Erlösdifferenz von 12.244 € zugunsten des Slow-Tracks (Tab. 8).

**Table 8:** 

	Gesamterlös
Fast-Track	25.384 €
Slow-Track	37.628 €
∆	12.244 €

### Patientenfall 1

Bei einer Verweildauer von 32 Tagen lag der Gesamterlös bei 25.384 €. Dieser errechnete sich aus dem DRG-Erlös von 19.084 € (DRG I03A), dem Pflegeerlös von 6247 € sowie einem Zusatzentgelt von 52,50 € für den Corona-Test (Tab. 6*).*

### Patientenfall 2

Hier lag der Gesamterlös mit 37.628 € für beide stationäre Aufenthalte deutlich höher als beim Fast-Track (Tab. 7). Für den ersten Aufenthalt (VWD 21 Tage) ergab sich aus dem DRG-Erlös von 19.084 € (DRG I03A), dem Pflegeerlös von 4099 € und dem Zusatzentgelt von 52,50 € für den Corona-Test ein Gesamterlös von 23.236 €. Beim zweiten Aufenthalt (VWD 9 Tage) konnte ein Gesamterlös von 14.392 € berechnet werden bei einem DRG-Erlös von 11.722 € (DRG I04Z), einem Pflegeerlös von 1417 € und den Zusatzentgelten von 52,50 € für den Corona-Test und 1200 € für die modulare Prothese (ZE 2020-25).

## Diskussion

Die Erlössituation bei der Behandlung periprothetischer Hüftinfektionen ist aufgrund der unzureichenden Abbildung im bisherigen DRG-System nicht angemessen und stellt für die Kliniken eine große ökonomische Belastung dar [11, 14], da die anfallenden hohen Behandlungskosten durch die Fallpauschalen des DRG-Systems nicht ausreichend gedeckt werden und dennoch von den Kliniken getragen werden müssen [2, 15, 17, 27].

Die vorliegende Arbeit hatte zum Ziel, die Erlössituation für periprothetische Hüftinfektionen für das seit dem 01. Januar 2020 geltende aG-DRG-System darzustellen. Durch Fallsimulationen einer periprothetischen Hüftinfektion sowie durch den Vergleich von zwei realen Behandlungsfällen des Jahres 2020 konnte gezeigt werden, dass beim Slow-Track mit langem prothesenfreiem Intervall generell ein höherer Erlös als beim Fast-Track mit kurzem Interimsintervall erzielt wurde, indem beim Slow-Track unter Umgehung einer Fallzusammenführung der Gesamterlös aus zwei stationären Fällen berechnet wurde. Beim Fast-Track hingegen konnte nur ein stationärer Fall abgerechnet werden.

In den simulierten Fallbeispielen ohne weitere Nebendiagnosen lag der Gesamterlös beim Slow-Track um 3586 € höher als beim Fast-Track, wobei derartige Fälle im klinischen Alltag eher die Ausnahme darstellen und in der vorliegenden Arbeit vor allem als Vergleichssimulation aufgeführt wurden.

In den simulierten Fällen mit zusätzlichen Nebendiagnosen zeigte sich eine große Erlösdifferenz von 13.416 € zugunsten des Slow-Tracks. Diese Fälle spiegeln die Realität wider, da Patienten mit periprothetischen Hüftinfektionen meist multiple Komorbiditäten aufweisen und einen hohen Pflegeindex haben [18]. Gerade bei multimorbiden Patienten wäre eine Fast-Track-Behandlung mit kurzem prothesenfreiem Intervall von 2–4 Wochen in einem stationären Aufenthalt durchaus vorteilhaft. Eine zwischenzeitliche Unterbringung der Patienten, die häufig in Form einer Kurzzeitpflege erfolgt, und sowohl Kliniken, Kostenträger als auch die Patienten selbst teilweise vor große organisatorische und finanzielle Herausforderungen stellt, wäre damit vermeidbar. Weitere Vorteile des Fast-Track-Konzeptes sind die Verkürzung der Gesamtbehandlungsdauer und die Reduktion der Gesamtkosten pro Behandlungsfall [20].

Durch die Analyse der realen Behandlungsfälle konnte der ökonomische Vorteil einer Slow-Track-Behandlung im aG-DRG-System unterstrichen werden, indem auch hier aufgrund des langen Intervalls von 11 Wochen eine Fallzusammenführung verhindert und zwei stationäre Behandlungsfälle abgerechnet werden konnten. Somit resultierte ein deutlicher Erlösunterschied von 12.244 € inklusive der abrechenbaren Zusatzentgelte zwischen Fast-Track und Slow-Track, wobei in beiden Fällen therapiebedürftige Nebendiagnosen bei nahezu gleich langer Verweildauer vorlagen.

Als weiteres wesentliches Ergebnis unserer Arbeit konnten wir mittels Fallsimulation zeigen, dass die zusätzlich kodierten Nebendiagnosen beim Fast-Track keinen Einfluss auf den DRG-Erlös hatten, sich jedoch beim Slow-Track in beiden Aufenthalten erlössteigernd auswirkten und zu einem höheren DRG-Erlös führten. Dies ist ein Hinweis darauf, dass eine periprothetische Hüftinfektion bei multimorbiden Patienten mit Fast-Track-Behandlung auch im aG-DRG-System nicht adäquat abgebildet wird, sodass dieses Konzept wegen der enormen Kostenbelastung und der unzureichenden Erstattung für die Kliniken nicht profitabel ist. In einer früheren Arbeit von Lieb et al. konnte durch eine Kosten-Erlös-Analyse gezeigt werden, dass beim Fast-Track eine deutliche Kostenunterdeckung im Vergleich zum Slow-Track resultiert [20]. Daher bedarf es hier einer Anpassung des Systems, um eine Fast-Track-Behandlung für die Kliniken aus ökonomischer Sicht attraktiver zu machen.

## Limitationen

Einschränkend muss gesagt werden, dass ausschließlich eine periprothetische Hüftinfektion mit Nachweis eines multisensiblen Staphylococcus aureus (MSSA) simuliert wurde. Andere Erregerspektren, wie multiresistente Keime, oder Infektionen ohne Keimnachweis blieben unberücksichtigt. Dies wäre zusätzlich zu untersuchen, um die Heterogenität der periprothetischen Infektionen abzubilden. Es wurde rein die Erlössituation beim zweizeitigen septischen Hüftprothesenwechsel dargestellt, ohne die jeweiligen Gesamtbehandlungskosten (Personal- und Sachkosten) für Fast-Track und Slow-Track zu berücksichtigen. Zur Ermittlung einer positiven bzw. negativen Kostendeckung sollte daher eine Kosten-Erlös-Analyse folgen.

## Fazit für die Praxis

- In dieser Arbeit konnten wichtige Einflussfaktoren auf den Erlös beim zweizeitigen septischen Hüftprothesenwechsel im aG-DRG-System (aG-DRG: ausgegliederte German Diagnosis Related Groups) dargestellt und deren unterschiedliche Auswirkung beim Fast-Track und Slow-Track analysiert werden.
- Im Fast-Track-Modell mit der Gesamtbehandlung des Falls inklusive der Reimplantation der Prothese in einem einzigen stationären Aufenthalt werden Kliniken vor allem bei multimorbiden Patienten finanziell deutlich schlechter gestellt als beim Slow-Track mit zwei separaten stationären Aufenthalten. Hierdurch wird das Fast-Track-Verfahren in seiner praktischen Umsetzung sehr benachteiligt.
- Daher gilt es, bei der Behandlung periprothetischer Hüftinfektionen durch Vermeidung ökonomischer Fehlanreize eine Veränderung und Anpassung der Erlössituation seitens der Kostenträger zu bewirken.

## Abkürzungen

aG-DRG: Ausgegliederte German Diagnosis Related Groups
CC: Komplikationen oder Komorbiditäten
COVID-19: „Coronavirus disease“ 2019
DRG: Diagnosis Related Groups
G‑DRG: German Diagnosis Related Groups
ICD: International Statistical Classification of Diseases and Related Health Problems
LBFW: Landesbasisfallwert
MDC: „Major Diagnostic Category“
MSSA: Multisensibler Staphylococcus aureus
mVD: Mittlere Verweildauer
ND: Nebendiagnosen
OGVD: Obere Grenzverweildauer
OPS: Operationen- und Prozedurenschlüssel
PCCL: Patient Clinical Complexity Level
SARS-CoV 2: „Severe acute respiratory syndrome coronavirus type 2“
TE: Transfusionseinheit
TEP: Totalendoprothese
UGVD: Untere Grenzverweildauer
VWD: Verweildauer
ZE: Zusatzentgelt
